# Allogeneic HSCT in Adolescents and Young Adults With Primary Immunodeficiencies

**DOI:** 10.3389/fped.2019.00437

**Published:** 2019-10-24

**Authors:** Emma C. Morris, Michael H. Albert

**Affiliations:** ^1^Institute of Immunity and Transplantation, University College London, London, United Kingdom; ^2^University College London Hospital and Royal Free London Hospitals, London, United Kingdom; ^3^Department of Pediatrics, Dr. von Hauner Children's Hospital, University Hospital, LMU Munich, Munich, Germany

**Keywords:** primary immunodeficiencies, PID, allogeneic hematopoietic stem cell transplantation, Allo HSCT, adolescents and young adults (AYAs)

## Abstract

Significant advances in hematopoietic transplantation over the past 20 years, have facilitated the safe transplantation of older adults with higher co-morbidities. In pediatric practice these advances have simultaneously improved outcomes for sicker children with complex, rare diseases including the primary immunodeficiencies, PID. With more widespread adoption of genetic sequencing, older patients with disease-causing mutations restricted to the hematopoietic system can be identified who may benefit from allogeneic hematopoietic stem cell transplantation (Allo-HSCT). Here we discuss the evidence for Allo-HSCT in adolescent and younger adults (AYAs) with PID.

## Introduction

Adolescents and young adults (AYAs) have specific medical and psychosocial needs that differentiate them from pediatric patients and older adults. In the setting of primary immunodeficiencies (PID), their clinical presentation is often atypical compared to infants or younger children, despite having the same underlying disease. In the oncology setting these differences have resulted in poorer outcomes and less unified approaches to medical care (1, 2), including in the setting of allogeneic hematopoietic stem cell transplantation (HSCT) (3).

The earliest reported HSCT for primary immunodeficiencies (PID) were performed some 50 years ago (4, 5). For the last 30 years, transplantation has been considered “standard of care” and the major therapeutic option for children with inherited cellular immunodeficiency disorders. Early HSCT is particularly important in infants or children presenting with serious or life-threatening infections, as without definitive treatment, patients with severe PID, such as severe combined immune deficiency (SCID), rarely survive beyond 1 year of age. In addition, younger patients have less end-organ damage from repeated or severe infections (6, 7). Indeed, historically, overall survival has been shown to fall from 95% in SCID patients transplanted at ages <3.5 months to 76% in older children (8, 9).

Although early HSCT for PID is preferable, this is not always possible. The clinical phenotype of “non-SCID” forms of PID can be very heterogeneous due in part to the high number of genetic and functional defects affecting T-, B-, NK-cells, neutrophils, and/or antigen presentation. This, together with a variety of other factors may result in patients surviving to adolescence or adulthood without HSCT, for example, less severe disease manifestations in childhood and/or later presentation, delayed diagnosis, lack of a genetic diagnosis, or a previous lack of a suitable donor (10).

In addition, until very recently, AYAs with PID have not routinely been considered candidates for HSCT. This reflected a lack of published data and a presumption that outcomes post-transplant would be inferior to that observed with younger patients due to increased transplant related mortality (TRM) and a higher risk of graft vs. host disease (GVHD).

Significant advances in hematopoietic transplantation over the past 20 years including refinement of HLA-tissue typing, adoption of reduced intensity conditioning (RIC) regimens, increased availability of alternative stem cell sources, improved methods of GVHD prophylaxis and improved supportive care have translated into better outcomes today compared to the early experience (6, 11).

This chapter aims to summarize the increasing evidence that carefully selected AYAs with PID can achieve equivalent outcomes following HSCT compared to that routinely achieved in pediatric cohorts.

## Indications for HSCT

PID patients referred for HSCT in adolescence or later typically have suffered a life-threatening complication of their immunodeficiency or have serious, treatment-refractory complications.

These may include: Bone marrow failure requiring long-term blood, platelet or cytokine support due to the high risk of (i) uncontrolled infection or bleeding, (ii) transfusion-associated iron overload affecting liver or cardiac function, or (iii) alloimmunization; PID-associated malignancy; Secondary hemophagocytic lymphohistiocytosis (HLH); Vital organ complications (e.g., to the kidney, lung, and gut) as a result of their PID, which have failed to respond to alternative treatments and where delay will lead to irreversible organ injury, thus precluding future transplant or significantly increasing the risk of complications.

In pediatric practice, “well” infants/children with a diagnosis of severe PID are typically offered HSCT prior to the development of significant infections, autoimmunity, and/or autoinflammation in order to reduce the risk of peri-transplant complications. Where the TRM risk is small and the severity/prognosis of the underlying disease is well-understood and predictable the “pre-emptive” use of HSCT is justifiable, examples of specific PID in this category include chronic granulomatous disease (CGD), Wiskott-Aldrich Syndrome (WAS), primary HLH and GATA2 Deficiency. However, for the few patients remaining well until late adolescence this risk-benefit balance is less clear, particularly for PID subtypes with less well-studied alloHSCT outcome. Studies that address the long-term prognosis of these patients are very difficult to perform, but natural disease outcome data are desperately needed to be able to determine the optimal role of HSCT (12). There is an urgent need for additional data from collaborative multicentre studies to determine the specific PID subtypes for which AlloHSCT confers genuine benefit in terms of both medical outcome and improvements in quality of life, when performed in adolescence and young adulthood. A number of such studies are in progress, including a large retrospective study co-ordinated through the Inborn Errors Working Party of the European Society of Blood and Marrow Transplantation (EBMT IEWP).

PID subtypes for which further data is required to determine the appropriate time to intervene with alloHSCT include amongst others XIAP deficiency, CD40L deficiency, genetically undefined combined immunodeficiency and late-onset SCID/hypomorphic RAG. Published outcome data for adult patients with complex CVID have indicated poorer outcomes than for other PID (13) and prospective studies are required for this indication.

As with specific PIDs presenting earlier in childhood, those diseases with extra-haematopoietic manifestations (e.g., ADA-SCID, Chediak Higashi, STAT3-HIES, etc.) will not be fully corrected with HSCT even if successful and alloHSCT should only be considered in adolescence or early adulthood if it is predicted to improve the patient's quality of life and/or survival.

## Role of Genetic Diagnosis

In patients where a genetic diagnosis has been confirmed and the mutation is known to be both pathogenic and haematopoietically restricted, HSCT or gene therapy are the only curative therapies and clearly indicated if the patient is otherwise eligible for transplant with sufficient renal, liver, cardiac, and pulmonary function. However, the recent widespread adoption of whole genome sequencing (WGS) in older patients has highlighted the complexity of making treatment decisions for patients with overt clinical disease in the presence of single or multiple genetic variants of uncertain significance (VUS) and/or heterozygous mutations in known PID genes. Sequencing information in the wider family is not always available when index patients are older.

Highly penetrant autosomal dominant monogenic PIDs are more likely to present at an early age, increasing the relative proportion of older patients having undefined or uncharacterized single or multiple genetic abnormalities.

## Conditioning Regimens

As with pediatric practice a wide variety of regimens are in use, but most experience to date has been with Flu/Bu, Flu/Mel, or Flu/Treo based reduced intensity conditioning (RIC) incorporating serotherapy (Alemtuzumab or ATG) for *in vivo* T cell depletion. Patients surviving to adolescence typically have residual functional cellular immunity necessitating conditioning to permit engraftment of allogeneic stem cells and prevent graft rejection, as a consequence unconditioned transplants are not indicated.

The use of post-transplant cyclophosphamide (PTCy) or αβTCR depletion to remove alloreactive T cells has facilitated the use of haploidentical donors in older recipients, without prohibitive risks of GVHD and/or graft rejection. However, the cumulative experience transplanting AYAs with haploidentical donors remains small, particularly in patients over the age of 18 years.

## Donor Selection

Preferred stem cell donors are 12/12 (or 10/10), CMV sero-matched, unaffected related donors in order to minimize TRM and risk of GVHD. However, most series published to date include large numbers of MUD and 1 Ag MMUD transplants, with good results.

At present there is insufficient published data in older PID patients (AYAs and older adults) to support the use of haploidentical donors in preference to less well-matched MUDs (2 Ag mismatch or less). In pediatric practice T-cell replete haploidentical transplants with PTCy have achieved good results for patients with PID and other inborn errors, where no matched donors are available (14). In non-PID settings, a number of prospective RCTs are being planned to determine the safety and efficacy of MMUD vs. Haplo in patients >18 years at HSCT.

## Optimal Timing of HSCT

The largest published series of HSCT for PID are pediatric, with the overwhelming majority of transplanted patients being <5 years old at the time of transplant. However, there is clear evidence within the pediatric setting that outcomes are better for younger recipients (15, 16). This, in part, reflects the clinical status of the patient at transplant. The shorter the time from onset of clinical symptoms to transplant, the lower the risk of developing resistant or refractory infections (bacterial, viral, or fungal) and end organ damage due to uncontrolled inflammation or autoimmunity. In AYA patients the number of co-morbidities are typically higher at transplant.

Recent data have indicated that the HCT-CI score (a validated co-morbidity index predicting high risk patients for HSCT in the setting of hematological malignancies) has predictive value for patients with PID (17). However, this study analyzed outcomes for mostly pediatric PID patients and needs to be validated in AYA and adult PID populations.

Where possible control of autoimmunity or inflammation should be achieved prior to transplant. PID-associated malignancies should be treated and in remission or VGPR as per routine practice in HSCT for lymphoid malignancies. For patients with EBV handling disorders, the inclusion of rituximab in the conditioning regimen can bridge the gap until functional immune reconstitution is achieved post-transplant.

## Outcomes

Historically, outcome data from the small numbers of PID patients aged >15 years at the time of HSCT have mostly been included in much larger pediatric series, making it difficult to interpret the results for this small subgroup. However, in the last decade there have been 12 published manuscripts describing outcome for larger numbers of AYAs, either in isolation or in combination with younger patients (13, 18–27). The total number of patients aged >18 years included in these studies was 154. These publications are summarized in Table 1.

**Table 1:** Summary of published Allo-HSCT outcome data for adolescent and young adult PID patients.

**References**	**Patient (no)**	**PID subtype**	**Age at HSCT years (range)**	**Donor (no)**	**Conditioning**	**OS**	**EFS**	**TRM**	**Engraftment**	**Median follow-up (range)**
Albert et al. (18)	18	6 CGD 12 other PID	18 years (15–22)	MRD (2) MUD (5) 1 Ag MMUD (11)	Full Bu/Cy (2); Full Bu/Flu (1); Sub Bu/Flu (7); Flu/Mel ± TT (1); Treo/Flu ± TT (7). All had serotherapy.	94%	94%	6%	100%	5 years (2–9 years)
Fox et al. (19)	29	11 CGD 18 other PID	24 years (17–50)	MRD (11) MUD (13) 1 Ag MMUD (5)	Flu/Mel/Alemtuzumab (20) Flu/Bu/ATG (8) Flu/Bu/Alemtuzumab (1)	89% at 1 year 85% at 3 years	90%	14%	100%	3.5 years (4 months−12 years)
Jin et al. (20)	8	Primary HLH	25 years (18–54)^*^	HaploID (6) MUD (2)	TBI/VP16/Cyclo (6); VP16/Flu/Bu/ATG (2)	88%	ns	12%	100%	27 months
Leiding et al. (21)	5 (AYA) 10 (ped <12 years)	STAT1 GOF	29 years (18–35) 8 years (1–17)	MRD (4); MUD (8); MMUD (1); HaploID (2)^#^; UCB (2, 2^#^)	Flu/Mel/Alem (4); Bu/Cy (3); Flu/Bu or Treo/ATG or Alem (6); Various other (4^#^)	20% at 1 year (AYA) 60% at 1 year (ped) 40% at 1 year (all)	ns ns ns	ns	50%^∧^	ns
Parta et al. (22)	17 (AYA) 20 (ped)	CGD	24 years (18–32) 8 years (4–17)	MRD (6); MUD (30); MMUD (1)	Bu/Alem (6); Bu/TBI/Alem (31)	82.5% (all)	80% (all)	17.5% (all)	85% (all)	3.4 years (range ns)
Shah et al. (23)	7	DOCK8 Defic	20 years (7–25)	HaploID (7)	Flu/Bu/Cyclo + low dose TBI (7)	86% (all)	ns	14%	100%	2 years (3 months−5.7 years)
Fu et al. (24)	30	1° HLH; EBV- HLH; Tu-HLH; Undef- HLH	23 years (14–52) 19 years (14–55) 24 years (14–44) 29 years (16–32)	HaploID (23); MRD (6); MUD (1)	Bu/Cyclo/VP16 (6)—for MRD TBI/Cyclo/VP16 + ATG (24)—for Haplo/MUD	63.3% at 2 year (100% in 1° HLH; 64% in EBV-HLH; 17.7% in Tu-HLH; 25% in Undef)	ns		100%	26 months
Wehr et al. (13)	14 (adult) 11 (ped)	CVID	34 years (18–50) 14 years (8–17)	MRD (14); MUD (10); MMUD (1)	BCNU/Flu/Mel (5); Flu/Mel (7); Flu/Mel/Treo (2); Flu/Bu ± TT (4); Bu/Cy ± AraC (6); Flu/Cy (1)	57% (adults) 52% (all patients)		44% at 1 year (all)	79% (adult)	ns
Grossman et al. (25)	14	GATA2 Defic	33 years (15–46)	MRD (4); MUD (4); UCB (4); HaploID (2)	Flu/low dose TBI (8); Flu/Cy/low dose TBI (6).	57% (all)	ns	28%	100%	3.5 years (1–5 years)
Güngör et al. (26)	13 (adult) 43 (ped)	CGD CGD	21 years (18–39) 9 years (0.8–17)	MRD (21); MUD (25); MMUD (10)	Flu/Bu ATG or Alemtuzumab (all).	92% (adult) 96% (all)	91% (all)	7%	93% (adult)	21 months
Spinner et al. (27)	21	GATA2 Defic	ns (15–49 years)	ns	ns	72% at 1 year; 65% at 2 year; 54% at 4 year	ns	ns	ns	14 months (0–180 months)

* Age at diagnosis; ns, not stated;

∧ secondary graft failure;

# *as second or third procedure; + Tu- tumor associated HLH, Undef, undefined HLH; adult = ≥18 years at transplant*.

### Overall Survival

In the majority of published series, the overall survival was equivalent to that achieved in younger children and infants being in excess of 80% at a median follow-up of between 14 months and 5 years (18–20, 22–24, 26). The studies reporting poorer outcomes, included a small multicentre series including 4 AYA patients with STAT1 GOF mutations and associated HLH (21), a large retrospective series of CVID patients, which included 14 patients aged 18–50 years with an OS of 57%, with 21% graft failure and 21% severe GVHD (13), and two separate series of patients with GATA2 deficiency, the first included 14 patients >18 years achieving an OS of 57% at a median follow-up of 3.5 years where a high rate of serious infectious complications was observed at 65% (25), and a single center US study of patients with GATA2 deficiency (*n* = 21 transplanted patients, OS at 2 years 65%) (27).

### Event Free Survival

The concept of event free (or disease free) survival is critical in HSCT for non-malignant disease, particularly when patients are being transplanted to prevent further disease progression and future life-threatening complications. The use of composite endpoints such as GVHD-free, relapse-free survival (GRFS), which were originally developed for GVHD studies (28), and are now being evaluated as predictors of longer-term OS, should be included in future transplant studies in the PID and non-malignant setting.

### Engraftment

The specific cellular defect associated with a given PID has a direct impact on the risk of graft failure. For example, in some forms of PID associated with immune dysregulation or significant autoinflammatory manifestations the risk of graft rejection is higher than that observed transplanting patients of a similar age with hematological malignancies. In the published literature reviewed here, 11 of the studies describing a total of 141 AYA patients reported on engraftment in detail. Typically, rejection was defined as <10% donor cells in the presence of disease recurrence. In six studies no graft failure or rejection was observed (18–20, 23, 24), despite a wide variety of transplant indications, conditioning regimens and donor source. In four studies the proportion of graft failures ranged from 8 to 50%, with a higher number of secondary graft failures (*n* = 6) than primary graft failures (*n* = 3). Of note, the rates of graft failure were higher in patients with preserved T-lymphocyte function, such as those with STAT1 GOF mutations: 50% secondary graft loss (21); CGD: 8–18% graft failure (22, 26); and complex CVID: 21% total graft failure including primary and secondary (13).

As with HSCT for hematological malignancies repeat transplant, CD34^+^ selected “top up” or donor lymphocyte infusions may be indicated in cases of graft failure or rejection. Management will vary on a case by case basis, informed by rate of change of multilineage chimerism and the presence or absence of cytopenias. For example, patients achieving stable mixed chimerism, normal peripheral blood counts, and independence from immunoglobulin replacement therapy do not require intervention if they remain free of recurrent infections or significant autoimmunity.

### Chimerism

The achievement of multi-lineage full donor chimerism is not in all PID a pre-requisite for sustained correction of the clinical phenotype post-transplant. Due to small numbers of patients, heterogeneity of underlying PIDs and variation in conditioning regimens, none of the published data in the AYA group has demonstrated a correlation between mixed donor chimerism and age of patient, underlying diagnosis or donor type. In the studies reported here, rates of multilineage full donor chimerism were between 48 and 94% at last follow up (18–20, 23–26).

### Immune Reconstitution

Very few published studies have reported on detailed immune reconstitution in all patients. In practice, detailed immune reconstitution data is not always available and “effective clinical immune reconstitution” can be considered to have occurred in patients who are infection-free, off immunoglobulins, successfully re-vaccinated and not requiring antimicrobials other than routine prophylaxis. In the published studies, including AYA patients reporting on immune reconstitution, >89% of patients had ceased immunoglobulin replacement therapy at last follow up. As predicted, these patients had achieved a high degree of donor B cell chimerism (18, 19, 26).

### Post-transplant Infectious Complications and Transplant-Related Mortality (TRM)

The rate of post-transplant infectious complications in AYAs with PID is higher than that observed in younger pediatric patients or in adults undergoing HSCT for non-PID indications. This likely reflects the greater infectious burden pre-transplant and in some cases the development of antimicrobial resistance, and is also reflected in the causes of TRM. Interestingly, not all AYA PID HSCT studies have reported specifically on TRM. Where reported, TRM was very low at 6% for 18 AYA patients with a median age of 18 (range 15–22.1) years at transplant (18) and still very acceptable at 14% for an older cohort with a median age of 24 years at transplant (range 17–50) (19). Causes of death were multi-organ failure secondary to sepsis (*n* = 2), granulomatous meningoencephalitis (*n* = 1), sepsis in the context of extensive chronic graft-vs.-host disease (*n* = 1) and disseminated adenovirus (*n* = 1). The widespread use of quantitative PCR for monitoring of EBV viremia and prompt management with rituximab prevented any PTLD deaths.

### Graft-vs.-Host Disease (GvHD)

The incidence of GVHD increases with age of recipient, degree of HLA disparity between donor and recipient and the use of non-T-lymphocyte (cell) depleting (TCD) conditioning regimens (29, 30). Therefore, in the AYA population, higher rates of GVHD are observed than that seen in much younger children and infants. Due to the almost universal use of serotherapy for TCD, most published patients experienced grades I-II acute GvHD (13, 18–21, 23–26). The incidence of severe, grades III-IV acute GvHD was between 3 and 21%. Similarly, extensive or severe chronic GVHD was only observed in up to 21% of recipients. In the CVID patient cohort (13) GVHD was a contributory factory for death in 88% of the patients developing severe GVHD (defined as grades III-IV acute GVHD or chronic extensive GVHD). Of note, single agent GVHD prophylaxis was used in these patients and their median age at transplant was 25 years. In up to date transplant practice, multiple agents in combination are typically used for GVHD prophylaxis either with or without PTCy (31) and T cell depleting serotherapy. Recent publications have also demonstrated that single agent PTCy can be effective as GVHD prophylaxis in pediatric and adult cohorts with non-malignant and malignant indications for HSCT (32, 33).

Once GVHD occurs following HSCT for PID, it should be treated aggressively in order to maximize response as there is no anti-leukemic benefit to the recipient. Conversely, when HSCT is performed for malignancy, the presence of chronic GVHD confers a reduction in relapse risk due to the concurrent graft-vs.-malignancy effect secondary to recipient directed alloreactivity from donor derived immune cells. Management of GVHD in PID patients is no different to other patients, but careful monitoring of pre-existing or latent infections (e.g., EBV, CMV, HPV, and aspergillus) is essential. Pre-transplant patients need to be carefully counseled regarding the risk of GVHD. If chronic extensive GVHD occurs involving skin, gut and/or lung the symptoms may mimic the underlying immune dysfunction for which the patient underwent HSCT and there may be no significant improvement in quality of life.

## Specific Challenges

### Fertility

Prior to HSCT concerns regarding the impact on fertility are common in adolescents and young adults. For both males and females, post-HSCT fertility is dependent on age of the patient, mechanism of action of the chemotherapeutic agent and dose delivered (34).

There is a wide variation in gonadotoxicity of different chemotherapeutic agents, but alkylating agents (busulfan and melphalan) cause dose-dependent, direct destruction of oocytes and follicular depletion, and may bring about cortical fibrosis and ovarian blood-vessel damage resulting in premature ovarian failure. For boys and young men after chemotherapy treatment, sperm production slows down or ceases altogether. Some sperm production usually returns in 1–4 years, but it can take up to 10 years. In post-pubertal boys, sperm should be stored prior to HSCT. In girls, sufficient time should be allowed pre-transplant for fertility preservation including egg/embryo collection and cryopreservation.

Post HSCT young women should be managed by specialist teams to prevent complications of primary ovarian failure and early menopause. Female patients wishing to have children post HSCT may use cryopreserved eggs for IVF with or without preimplantation genetic diagnosis. It should be noted that the use of preimplantation genetic diagnosis has ethical and religious implications and is not available or accepted for all patients in all countries. However, for all patients with poorly characterized or autosomal dominant genetics, detailed genetic counseling is required to discuss the risks of giving birth to an affected child.

### Psychosocial

Especially in the AYA population timing of transplant, where possible, should take into consideration schooling, further education, employment, and family commitments. For many patients the decision to finally proceed to transplant carries a heavy psychological burden. Even in the context of a chronic, severe, life-limiting condition, the prospect of “bringing forward” the risk of death by undergoing HSCT is difficult for the patient and family members. Compared to younger children, poorer compliance with post-transplant hospital visits or medication including infection and GVHD prophylaxis should be expected in AYAs.

## Future Approaches

For patients without a matched related donor or a 10/10 matched unrelated donor, the decision between using multiple mismatched cord units, a mismatched unrelated donor, a haploidentical donor or to consider gene therapy (where available, e.g., for X-SCID, ADA-SCID, WAS, X-CGD, and in development for other diseases including AR-CGD and CD40L deficiency) remains difficult, in part due to the relatively limited experience of all these modalities in older patients with PID. The use of alternative donors has a proven safety and efficacy record in younger children with PID and in adults with hematological malignancies. It is predicted that the use of PTCy and or αβTCR depletion in these older patients will also facilitate the safe use of HSCT in a wider group of potential recipients with alternative donors. Gene therapy has been successfully used in AYAs for WAS (35, 36) and X-linked CGD [manuscript submitted] where appropriately matched allogeneic stem cell donors were not available.

## Summary

Recent data has established that HSCT in AYAs with PID is safer than expected, that engraftment can be reliably achieved and overall survival is excellent for well-selected patients. Despite the availability of next generation gene sequencing for a large proportion of our patients, the decision to proceed to HSCT remains a clinical decision. The ability to transplant a patient does not always mean it is the right thing to do.

There is an urgent need to inform practice further with large international multicenter studies designed to assess outcome following HSCT for PID in adolescents and adults, together with equivalent studies describing the natural history of these rare diseases for un-transplanted patients. It is important that future prospective studies include detailed analysis of functional immune reconstitution, lineage-specific chimerism, GRFS, quality of life, psychosocial impact, and late effects.

## Author Contributions

All authors listed have made a substantial, direct and intellectual contribution to the work, and approved it for publication.

### Conflict of Interest

The authors declare that the research was conducted in the absence of any commercial or financial relationships that could be construed as a potential conflict of interest. The handling editor AG declared a collaboration with the authors EM and MA.
